# Nutraceutical properties of *Leucaena leucocephala, Manihot esculenta, Cajanus cajan* and a foliage blend in goat kids infected with *Haemonchus contortus*

**DOI:** 10.1038/s41598-020-66870-5

**Published:** 2020-06-19

**Authors:** Nathalie Minatchy, Carine Marie-Magdeleine, Miguel Garin, Ferdy Nimirf, Dimitri Romil-Granville, Lucien Philibert, Valeriuse Calif, Jean-Christophe Bambou, Harry Archimède

**Affiliations:** 1 INRA, UR143 Unité de Recherches Zootechniques, Domaine Duclos Prise d’eau, F-97170 Petit-Bourg, Guadeloupe; 2 INRA, UE1294 Plateforme Tropicale de recherche sur l’Animal, Domaine Duclos Prise d’eau, F-97170 Petit-Bourg, Guadeloupe

**Keywords:** Gastrointestinal diseases, Physiology

## Abstract

Protein and condensed tannin-rich foliage (TRF) are potentially useful as nutraceuticals. The main objective of this study was to evaluate the diet and anthelmintic properties of three TRF types both individually and in combination. We hypothesized that synergistic or antagonistic effects on feed and anthelmintic values related to associations between TRF types may occur. Nutritional and anthelmintic characteristics of *Leucaena leucocephala, Manihot esculenta, Cajanus cajan* and a mixture of the compounds were evaluated using alfalfa pellets as a control. TRF ingredients were combined with *Dichantium* hay (48 and 52% of dry matter intake respectively) in mixed diets were consumed by Creole goat kids. Measurements were carried out in animals without parasites and in animals artificially infected with *Haemonchus contortus*. Individual feed intake and the digestibility of each diet was measured along with kid growth. There were no significant differences between the growth rates of pre-infected animals and animals fed mixed diets that included alfalfa. A strong anthelmintic activity is observed with *Leucaena leucocephala* contrary to other TRFs. This work confirms variable dietary and anthelmintic properties of TRF. The combination of TRF did not have synergistic or antagonistic effects on feed value or the anthelmintic potential of TRF.

## Introduction

The fight for food security and considerations regarding the worsening of global warming have produced public opinion that is increasingly hostile to the use of synthetic chemical molecules in the food production chain, which prompted us to evaluate existing agro systems and farming practices^1^. To date, parasite control of small farmed ruminants is primarily based on the preventive or curative use of chemotherapeutics. Integrated control of gastrointestinal nematodes of livestock is an ongoing area of research. With this in mind, protein and condensed tannin-rich foliage (TRF), including legumes, are potentially useful nutraceutical compounds. However, although a large degree of variability with respect to the responses of animals have been reported in the literature^2,3^. Condensed tannins (CTs) and proteins directly and indirectly affect the development and effects of gastrointestinal nematodes (GINs). Nevertheless, the beneficial effects of condensed tannins against GINs can be offset by their depressive effect on food intake and digestion, feed value and, consequently, animal growth and/or milk production. In addition, CTs can also reduce methane emissions and ammonia volatilization within urine or dung^4^. Initially, dietary concentrations of CTs were suspected to explain the large degree of variability observed within animal responses to CTs in relation to dose-dependent anthelmintic^5^, methane emission^4^ and protein digestibility effects reported in the literature. Recent work has highlighted the importance of the molecular composition and structural traits of CTs, as well as of the diet containing CTs^2^. The main objective of this study is to evaluate the nutraceutical properties of *Leucaena leucocephala, Manihot esculenta, Cajanus cajan* pellets and a mixture of the three foliage types. These forages were chosen to have plants with a large gradient of CTs content. We also hypothesized that synergistic anthelmintic effects may result from intake a combination of TRF types. This last hypothesis is based on knowledge indicating that the two major components of CTs, procyanidin (PC) and prodelphinidin (PD) have variable distributions in plants and PD has been shown to have greater anthelmintic activity^2^. In addition, other phenolic compounds have also been shown to be unevenly distributed in plants with anthelmintic activity^2^. In addition, other secondary metabolites (hydrolysable tannins, saponins…) contained in TRFs could have anthelmintic activity.

## Results

The chemical compositions of types of feed are reported in Table 1. *Dichanthium spp* hay is characterized by its low crude protein (CP) levels and high fibre content, which indicates that it is a low quality grass. The foliage considered had higher CP content than alfalfa, except *Cajanus cajanus*, which had less CP. Intake and total tract digestion values of experimental diets are reported in Table 2. Diet intake was lower in kids provided hay compared to those provided mixed diets according to LW^0.75^ consumption values reported. Among the mixed diets considered, intake was lower for *Cajanus cajan* and mixed foliage, which was in line with the decreased hay intake observed in kids provided the diet.

**Table 1:** Chemical composition (g/kg), energy and protein value of diet ingredients: Organic matter (OM), Crude Protein (CP), Neutral Detergent Fibber (NDF), Acid Detergent Fibber (ADF), Acid Detergent Lignin (ADL), Condensed Tannin (CT), metabolizable energy (ME, kcal/kg) and metabolizable protein (MP).

	OM	CP	NDF	ADF	ADL	CT	ME	MP
*Dichanthium spp* hay	924	72	735	390	62	0	1674	42
Alfalfa pellet	893	179	313	219	56	0	2895	71
*Leucaena leucocephala* pellet	913	218	288	186	103	123	2574	82
*Manihot esculenta* pellet	910	205	463	327	166	77	2134	77
*Cajanus cajan* pellet	938	159	474	341	166	213	1949	65

**Table 2:** Mean values of dry matter (DM), organic matter (OM), Crude Protein (CP), Neutral Detergent Fibre (NDF) and Acid Detergent Fibre (ADF) intake and total tract digestibility (ttd) of *Dichanthium* hay or mixed diets of *Dichanthium* hay and alfalfa, *Cajanus cajan*, *Leucaena leucocephala*, *Manihot esculenta*, a blend (1/3 *Cajanus cajan*, 1/3 *Leucaena leucocephala*, 1/3 *Manihot esculenta*) of foliage as pellet fed by of Creole kids goats.

	Leucaena leucocephala	Manihot esculenta	Blend	Cajanus Cajan	Dichanthium spp	Alfalfa	SEM	P
**Intake**								
Dry matter g/d	455.1a	447.5a	404.0b	414.2b	312.4c	444.5a	14.50	0.001
Dry matter /g/LW^0.75^	62.6a	61.4a	56.3b	57.4b	41.2c	62.6a	1.81	0.001
Dry matter Hay g/d	270.5a	265.3a	224.1b	231.7b	312.4c	270.1a	13.60	0.001
Digestible Dry matter /g/LW^0.75^	38.1a	35.5b	30.5c	31.8c	27.3d	41.2a	0.90	0.001
**Total tract digestibility**								
Dry Matter (%)	60.4a	55.9b	53.5b	53.9b	61.9a	63.6d	1.78	0.001
Crude protein (%)	53.2a	48.9b	45.1b	44.8c	41.6c	58.5d	1.77	0.001
Neutral Detergent Fibre (%)	64.3a	62.9a	59.2b	60.5b	71.5c	67.9d	1.52	0.001
Acid detergent Fibre (%)	58.6a	52.8b	49.3c	50.8c	68.2d	64.3e	1.75	0.001

There was no depressive effect of the infection on intake. Observed variation (<10%) within diets week to week was similar to that observed for non-infested animals given the hay. These differences were due to the quality of the hay. There was also no significant effect of infestation on total tract digestion of diet components.

Based on a hypothesis of fixed total tract digestibility of Dry Matter (Dtt_DM_: 61.9%) and Crude Protein (Dtt_CP_: 41.6%) of *Dichanthium spp* estimated in this trial when hay was the only ingredient in the diet, the total tract digestibility of TRFs have been estimated. The estimated tract digestibility values (%) of the DM and CP for the ingredients *Leucaena leucocephala, Manihot esculenta*, *Cajanus cajan*, the TRF blend and alfalfa were 63.1 and 59.5, 52.4 and 53.1, 47.9 and 47.2, 48.7 and 46.0, and 70.9 and 69.5, respectively. The hierarchies observed for neutral detergent fibre (NDF) and acid detergent fibre (ADF) digestion followed the same trends as those observed for dry matter.

Details regarding nitrogen metabolism of kids fed the diets are included in Table 3. Nitrogen intake was lowest in kids fed hay and increased (from lowest to highest) in kids fed *Cajanus cajan*, the compound blend, alfalfa, *Manihot esculenta* and *Leucaena leucocephala*. There were no significant differences between diets with regard to urinary nitrogen excretion, even when diets were compared using equal nitrogen intake values. Faecal excretion of nitrogen, when equal quantities of nitrogen intake were compared, was significantly lower in kids fed alfalfa or *Leucaena leucocephala* compared with the others diets.

**Table 3:** Nitrogen balance, Average Daily Gain (ADG), Feed conversion with Creole kids fed *Dichanthium* hay or mixed diets of *Dichanthium* hay and alfalfa, *Cajanus cajan*, *Leucaena leucocephala*, *Manihot esculenta*, or a blend (1/3 *Cajanus cajan*, 1/3 *Leucaena leucocephala*, 1/3 *Manihot esculenta*) of foliage as a pellet.

	Leucaena leucocephala	Manihot esculenta	Blend	Cajanus cajan	Dichanthium spp	Alfalfa	SEM	P
N intake (g/d)	9.6a	9.0b	8.0c	7.3d	3.6e	8.1f	0.118	0.001
N faecal excretion (g/d)	4.7a	4.8a	4.6a	4.2a	2.2b	3.5c	0.162	0.001
N urinary excretion (g/d)	1.4a	0.9b	0.9b	0.9b		1.15a	0.169	0.1580
N retained (g/d)	3.6a	2.9b	2.4c	2.1c		3.5a	0.227	0.0001
N faecal excretion^a^, g/day	3.6a	4.1b	4.4b	4.4b	4.7b	3.3a	0.228	0.0001
N urinary excretion^a^, g/day	1.0	0.8	1.0	1.2		1.2	0.193	0.4807
ADG, g/day	70.7a	66.9a	60.6a	59.1a	26.4b	70.3a	10.78	0.0262
ADG^b^, g/day	120.8a	96.2b	80.3b	68.4c		119.2a	8.12	0.0001
ADG^c^, g/day	283.1a	259.6a	286.2a	281.7a	189.0b	281.1a	18.38	0.0109
Feed conversion, feed/kg ADG	8.1	9.0	12.1	9.8	13.2	9.0	2.23	0.3327

^a^Means were estimated by taking nitrogen intake as a covariate in the statistical analysis model. ADG estimated on the basis of energy intake^b^. ADG ^c^ estimated on the basis of N retained.

The average daily gain (ADG) values are reported in Table 2. ADGs were lower in kids fed hay while no significant differences were recorded between other diets. ADG values decreased with infestation. Post infestation values (except for hay diets consumed by uninfected animals) were 61.7, 22.7, 37.7, 47.0, 27.8 and 35.1 g/d for kids fed *Leucaena leucocephala, Manihot esculenta*, the compound blend*, Cajanus cajan*, hay and alfalfa diets, respectively.

**Figure 1:**
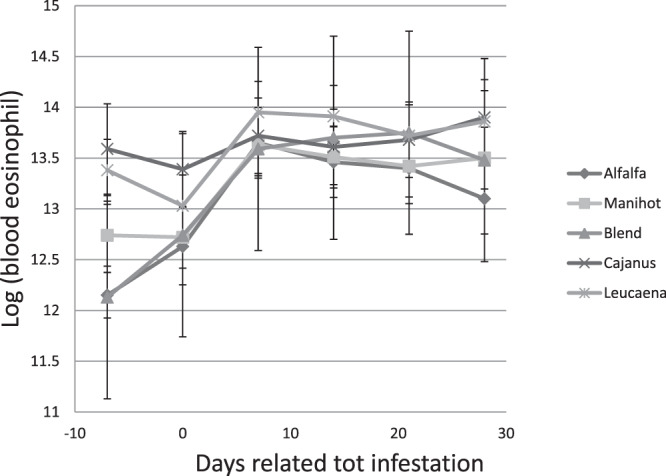
Eosinophil levels in the blood of Creole goat kids fed *Dichanthium* hay or mixed diets of *Dichanthium* hay and alfalfa, *Cajanus cajan*, *Leucaena leucocephala*, *Manihot esculenta*, or a blend (1/3 *Cajanus cajan*, 1/3 *Leucaena leucocephala*, 1/3 *Manihot esculenta*) of foliage as a pellet.

Weekly evaluations of eosinophils are summarised in Fig. 1. Overall, eosinophils increased after the infestation and peaked around the seventh day of infestation until they gradually returned to initial levels. There were no significant differences in levels of eosinophils observed between diets provided.

**Figure 2:**
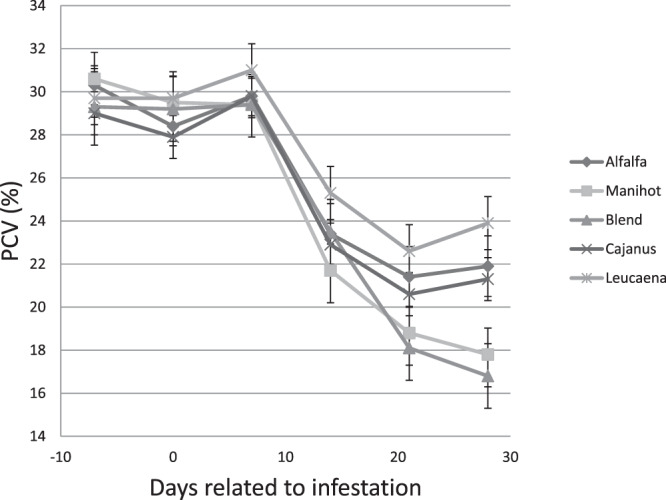
Packed Cell Volume (PCV) in the blood of Creole goat kids fed *Dichanthium* hay or mixed diets of *Dichanthium* hay and alfalfa, *Cajanus cajan*, *Leucaena leucocephala*, *Manihot esculenta*, or a blend (1/3 *Cajanus cajan*, 1/3 *Leucaena leucocephala*, 1/3 *Manihot esculenta*) of foliage as a pellet.

Weekly evaluations of blood packed cell volume (PCV) are summarised in Fig. 2. There were no significant differences between values before and after infestation. PCV tended to decrease on the fourteenth day after infestation and differences between diets were greatest 28 days post-infestation. Globally, PCV values were higher (P < 0.001) with *Leucaena leucocephala* compared to the other diets. PCVs values were lower (P < 0.001) in kids fed *Manihot esculenta* and the compound blend and compared to the other diets.

**Figure 3:**
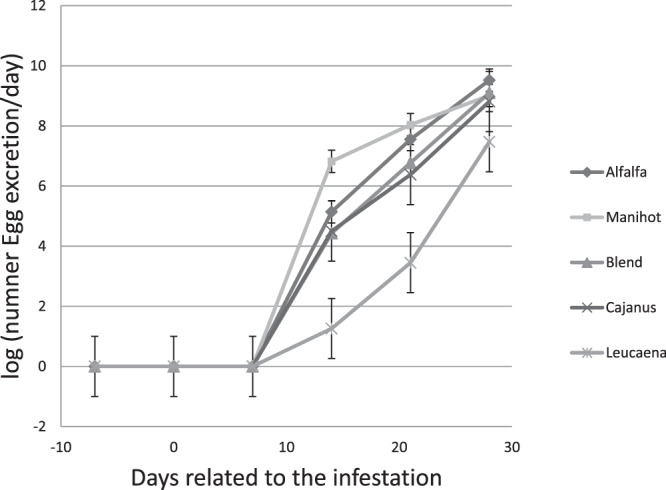
Faecal Egg Counts (FECs) of Creole goat kids fed *Dichanthium* hay or mixed diets of *Dichanthium* hay and alfalfa, *Cajanus cajan*, *Leucaena leucocephala*, *Manihot esculenta*, or a blend (1/3 *Cajanus cajan*, 1/3 *Leucaena leucocephala*, 1/3 *Manihot esculenta*) of foliage as a pellet.

The weekly evaluation of faecal egg counts (FECs) are summarised in Fig. 3. As expected, the FEC was zero for uninfected kids. FECs varied depending on duration of infestation and diet. The FEC values observed in kids fed *Leucaena leucocephala* were lower (P < 0.001) than those of kids fed other diets. There were no significant differences between *Manihot esculenta* and alfalfa diets, and kids fed the diets had significantly higher FEC values than those reported for *Cajanus cajan* and the TRF blend. The high FEC values observed in kids fed *Manihot esculenta* were associated with very high and suspicious FEC counts in two kids at day 14 post infection.

**Figure 4:**
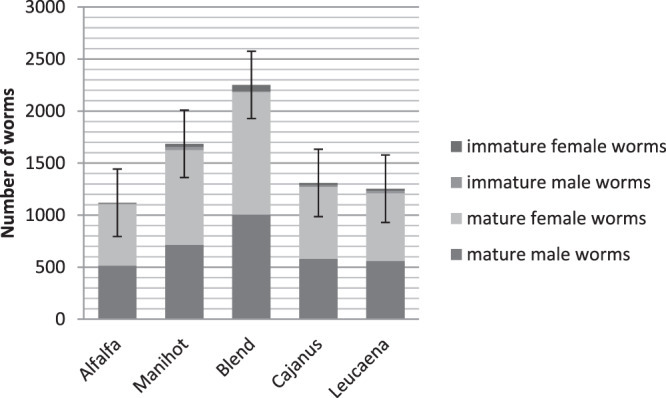
The number of worms in the abomasum of Creole goat kids fed *Dichanthium* hay or mixed diets of *Dichanthium* hay and alfalfa, *Cajanus cajan*, *Leucaena leucocephala*, *Manihot esculenta*, or a blend (1/3 *Cajanus cajan*, 1/3 *Leucaena leucocephala*, 1/3 *Manihot esculenta*) of foliage as a pellet.

No significant differences in any of the indicators considered were observed regarding the profiles of abomasum worm populations within kids (Fig. 4). The prolificacies of mature worms were 278, 4981, 502, 562, 6076 eggs for *Leucaena leucocephala, Manihot esculenta, Cajanus cajan* foliage, TRF blend and alfalfa respectively. Except for *Manihot esculenta*, the prolificacies tend to be similar (P < 0.15) with the TRF compared with alfalfa.

Data obtained from kids fed blended diets were consistent with those values obtained for each of the ingredients in the mixture, whatever the nutritional or health criterion.

## Discussion

The chemical compositions of feed provided to goat kids in experiments here are consistent with previously published data^6^. The low intake and total tract digestibility values associated with kids provided *Dichanthium spp* hay was consistent with data showing its chemical composition, which indicated that it was poor quality hay. Estimates of the total tract digestibility of TRF and alfalfa, obtained by difference from that measured on *Dichanthium spp* hay when it was the only ingredient in the diets, seems relevant. This is especially true when referring to estimates of the total tract digestibility of alfalfa, for which values are available in the literature. However, an overestimation of the total tract digestibility of TRFs cannot be eliminated. Indeed, hay digestion could be improved in kids provided mixed diets because TRFs by promoting microbial activity in the rumen, increases its fibrolytic potential (described below). Further, our data seem to indicate that the DM digestibility of TRF is highly variable (47.9 to 63.1%). None TRF-associated values reached values estimated for alfalfa, which was classified as high-value feed.

Considering estimated total tract digestion of DM and CP, diet containing *Cajanus cajanus* was classified as low-value feed, and *Leucaena leucocephala* and *Manihot esculenta* were classified medium-value feed. When the protein value of TRF was assessed on the basis of the total digestibility of CP, the same classification of *Cajanus cajanus*, *Leucaena leucocephala* and *Manihot esculenta* was determined. These results are consistent with theoretical degradability (DT_CP_) values of CP obtained in the laboratory using the same TRF. DT_CP_ values of *Leucaena leucocephala, Manihot esculenta* and *Cajanus cajan* were 64.5, 65.0 and 48.0, respectively. When Average Daily Gain (ADG) values were considered, slight differences in comparisons within TRF and between TRF and alfalfa were observed. First, differences in feed value between TRF and alfalfa would decrease. Within the TRFs, *Cajanus cajan* would have a lower feed value as illustrated with its metabalolizable energy and protein values. These differences in TRFs feed value are also illustrated by the ADGs registered between the diets. When comparing growths obtained on the basis of energy and protein intake from diets, energy would not be the first limiting factor in kid growth. The ADGs obtained with the nitrogen retention method are close to the real measured growth rates. We can therefore hypothesize that protein intake would the first limiting relatively to energy.

Depending on its nature, CTs could protect feed from microbial digestion in the rumen and enzymatic digestion in the intestine^7^, thus modulating the potential of TRFs to: i) provide fermentable nitrogen in the rumen; ii) alter the digestibility of proteins in the intestine. It is generally understood that the consumption of more than approximately 4–5% CTs, a value that varies according to the nature of the CTs, results in the reduced protein value of TRFs^2^. TRFs used in this experiment were above this 4–5% threshold, so proteins within the feed were partially protected from digestion. This can be seen when examining Dtt_CP_ values, which were lower than that of alfalfa. Nevertheless, Dtt_CP_ values were not exclusively related to CT concentrations with respect to the estimated digestibilities of different TRFs. However, effects of CTs may not be proportional to their concentrations, since the lowest digestibility values observed have not been associated with plants with the highest CT content. Previous works^7^ by other authors have shown that tannins likely produce high-threshold effects, which shou**l**d be taken into account.

Throughout experiments performed here, no external signs of intoxication (anorexia, ruminal atony, hepatic and renal failure, ulcers along the digestive tract, and severe gastroenteritis) were observed, irrespective of the type of TRF provided. These effects of intoxication have been reported in the literature, but have been mainly associated with plants rich in hydrolysable tannins^7^. Low intake has also been associated with TRF, which is a result of its astringent taste^7,8^. This was not observed in the present experiment where TRFs were 48% of the diet provided. The growth of Creole goats fed TRFs with alfalfa, with respect to growth potential values (about 80 g/d) and performance values, indicate that TRFs provided in these experiment are good supplements and improve the quality of low-quality forage diets. The rate of incorporation of TRFs into the mixed diets is an important factor when considering their use. Optimal rates of TRF incorporation vary depending on the experimental objective. For instance, enhancing the use low-value forage may require different quantities of TRF than maximizing animal performance. The use of foliage from browses and tree fodders to supplement low-quality roughage has been the subject of several reports^9–11^. Foliage provides nitrogen, minerals and vitamins are insufficiently provided by low-quality roughage. Consequently, it improves the activity of rumen microbes, which enhances fibre utilization. We assume the feeds in this experiment provided similar benefits. In our experiment, foliage contributed to an average of 48% of the total dry matter ingested. Patra^9^ reported that the positive effects of foliage on rumen microbes were obtained when foliage was at least 16% of the diet. Further, foliage levels up to 42% have the potential to enhance the performance of sheep fed low-quality roughage. Our results are in accordance with this report and indicate a positive effect of supplementation on animal growth. Although not significant, growth differences between diets provided may be explained by the potential of TRF to provide digestible protein within the intestine.

Hoste *et al*.^12^ assimilate GIN infections to a nutritional disease because of their major negative impacts intake and total tract digestibility of the diet, and the reorientation of nutrient use for the maintenance of tissue homeostasis. We did not observe any effect of animal infestation on feed intake and digestion. This result is in accordance with a previous report^13^. Intake and digestibility decreases have been observed in infested animals compared with healthy ones, but this finding has not been exclusively reported in the literature^13,14^. It is likely that differences in the history and health of animals, the infecting strain, the extent of the infection, and feed characteristics explain the variability observed in the literature^15^. Reductions in PCV, which were observed 14 d post-infection, were expected and reflected the haematophagous activity of *H. contortus*^16^. Cei *et al*.^13^ reported an 23.4% average reduction of PCV in goats for a one-unit increase in log-transformed FEC. Protein over nutrition with diets that increase the supply of digestible protein in the intestine, partially compensates for endogenous losses (blood, sloughed cells, etc.)^17^. PCV values determined here indicate that different TRF types have different capacities to compensate for these types of losses. These differences are not exclusively a result of differences in total digestible protein ingested in relation to the estimate made for each of the diets. We hypothesize that differences in amino acid profiles may also contribute to differential effects of TRF observed^18^. Condensed tannins inhibit rumen bacterial enzyme activity, bacterial growth and proteolysis in the rumen. Depending on the level of degradation of the feed protein and the availability of energy in the rumen for microbial protein synthesis, the ratio of Microbial Protein/ Feed Protein arriving in the rumen differed. Amino acid composition of microbial protein is relatively constant unlike dietary protein, which varies considerably^18^, although we do not have consistent data for TRFs. The amino acid profile of microbial protein would not be in balance with that of selected effect or molecules, thought to be involved with immune responses to gastro GINs^18^. Consequently, relatively larger quantities of microbial proteins would be required to meet these specific amino acid requirements^18^.

In this study, the reduction of FEC with the intake of TRF was only significant with *Leucaena leucocephala*, which is not the richest CT plant. Our results show that the anthelmintic activity varied according to the type of TRF provided as reported in literature^2^. These differences were not only proportional to concentrations of TC like reported in literature^19^. There are likely effects of specific tannin compositions that are not yet clear^2^. Other secondary metabolites in the plants could also have an anthelmintic activity^2^. Anthelmintic effects of TRF are associated with prodelphinidins and galloylated CT^2^. The TRFs used in this study have varying concentrations in these compounds as reported by Marie-Magdeleine *et al*.^20^. *Manihot esculenta* is richer in PD (PC/PD ratio = 0.16), which is quite different from *Cajanus cajan* (PC/PD = 0.85). *Leucaena leucocephala* has a more balanced composition (PC/PD = 0.59). The percentage of galloyl groups is very high for *Manihot esculenta* (37.44%) and *Leucaena leucocephala* (26.44%), whereas no galloyl group is found in *Cajanus cajan*.

The number and profile of worms (male, female, immature, mature) were not significantly different in kids fed different types of TRFs. However, the quantities of eggs excreted were affected. Based on previous studies, we had hypothesized that worm prolificacy could vary with diets. The depressive effect of CT on worm prolificacy has previously been reported^5,21^. Our data does not confirm these results. Really, if the highest prolificacy of worms is observed with the alfalfa diet that does not contain TC, there is no linear relationship between prolificacy and TC concentration of diets. The explanation for the data on worm proliferation is not obvious. Recent data show that eggs excretion may decrease as the population of worms in the abomasum increases^22^. This reduction in prolificacy is also an expression of the host animal’s resistance and/or resilience to GINs^22^.

Overall, whatever the indicator used to measure the anthelmintic activity of TRFs, there is a high variability of results as it is often the case in this type of trial where the number of animals is always limited for reasons of experimental logistics. Additional trials are therefore necessary to confirm the results obtained. Whatever the indicator considered the anthelmintic activity seems to be higher with *Leucaena leucocep*hala which is not the richest TRF in CT. It also has the best nutritional value which makes it a good nutraceutical. The relatively low activity of *Cajanus cajan* is quite surprising in view of its high CT content. The anthelmintic activity of the blend of TRFs which seems to be the average of that of TRFs indicates the lack of synergy between plants in this study.

## Conclusions

This work confirms dietary and anthelmintic properties of TRFs, which varied depending upon the type of feed provided. Providing mixed diets of TRFs to Creole kid goats, produced high levels of growth. However, the anthelmintic potential of TRFs was more variable. Combinations of TRFs did not have synergistic or antagonistic effects on the feed value or the anthelmintic potential of TRFs.

## Materials and methods

### Location

This research was carried out at the animal experimental station of the French National Agricultural Research Institute, Guadeloupe. This experimental unit has an accreditation to experiment (n°A971802) and involved staff has been trained in experimentation and animal welfare. Animals were treated in accordance with the guidelines and regulations for animal experimentation of the French Ministry of Agriculture. The protocol (APAFIS#5527–2016050608133139v2) was validated by the Higher Education and Research under the advice of the Animal Care and Use Committee of French West Indies and Guyana (N°069).

### Feed

The experiment was designed to evaluate nutritional and anthelmintic properties of *Leucaena leucocephala* (L), *Manihot esculenta* (M) and *Cajanus cajan* (C) foliage alone or in combination (B). These plants also contain different levels of condensed tannins, which were determined based on their HPLC profiles (Minatchy *et al*., unpublished data). Six diets were evaluated: Diet D, *Dichanthium spp* hay *ad libitum*; diet DA, D + 225 g/d of Alfalfa pellet; diet DL, D + 225 g/d of L pellet; diet DM, D + 225 g/d of M pellet; diet DC, D + 225 g/d of C pellet; diet DB, *Dichanthium* hay *ad libitum* + 225 g/d containing a mixed pellet containing 1/3 L, 1/3 M and 1/3 C. The alfalfa pellet was used in experiments as a foliage-rich, tannin-lacking control. The inclusion of mixed TRF sources was intended to detect possible synergies or antagonisms resulting from the association of TRFs on nutritional and anthelmintic values. Synergy and antagonism refers to event where two or more agents produce an effect greater or lower in combination than would be predicted from their individual contributions.

The hay used was mainly composed of 60-day-old *Dichanthium* spp derived from a fertilised and irrigated natural savannah grass. L foliage was from collections of 9- to 12-month-old fallow, not fertilised farmlands. M foliage was collected from 8- to 12-month-old crops during the harvest of tubers. C foliage was collected from 8- to 10-month-old crops post-harvest. The young stems and leaves of three plants were harvested and subsequently sun dried in a greenhouse for 3 to 5 d. Leaves with petioles were isolated from the stems and were ground and granulated. Alfalfa pellets came from industry. The composition of feed provided is shown in Table 1.

### Animals and experimental design

Forty-nine goat male kids aged approximately 4 mo. old, weighing on average 13 ± 2.0 kg at the beginning of the experiment, were used during a 3-month trial. The age and sex of goats used for the experiment meant animals were particularly sensitive to gastrointestinal strongyles. All the animals came from the INRA farm where they were raised in a pasture in accordance with current French welfare regulations. Before beginning the experiment all the kids were orally administered praziquantel (CESTOCURE, Bayer) and ivermectin (ORAMEC, Merial, Lyon, France, 0.3 mg/kg body weight). Faecal egg counts were performed after treatment to confirm the parasitic-free status of animals.

The experiment included four weeks of diet adaptation, 2 weeks of measurements before artificially infesting a portion of the kids, 4 weeks of post-infection measurements, the slaughter of kids 4 weeks post-infection. The kids were experimentally infected with a single oral dose of 10,000 *H. contortus* third-stage, infective larvae (L3). This dose was provided in order to produce very high levels of infection (based on faecal egg excretion), which were found in kids grown in the pasture.

Six experimental lots based on the diet and the parasitic status (infected *versus* uninfected) of animals were formed as follows: six uninfected kids fed diet D; nine uninfected then infected kids fed DA; ten uninfected and subsequently infected kids fed DL; ten uninfected and subsequently infected kids fed DM; seven uninfected and subsequently infected kids fed DC; seven uninfected and subsequently infected kids DB. The lots initially contained 10 animals. The numbers included in some were reduced because the pre-experimentation, anthelmintic treatment was not 100% effective. According to this experimental design, the pre-infestation period acts as experimental control.

The lots were balanced on the basis of kid weight and ADG between 30 and 90 d. Five animals per group were placed in metabolic cages for the collection of urine and the others in individual boxes on the ground. The kids were fitted with faecal bags in order to collect faeces. Reduced numbers of animals in lots DB and DC is a result of the availability of C foliage, which was lower than predicted. A kid was removed from lot 2 because it was not parasites free prior to artificial infestation.

### Measurements and calculations

Metabolizable energy (ME) and protein metabolizable (MP) values of feed were estimated as described by Salah *et al*.:^23^ ME (kcal/kg) = (−2.03 + 4.03 x DOM) x 10; MP (g/kg) = 22.6 + 0.256 x CP + 0.043 x DOM. All measurements of the characteristics of kids were carried out individually. Kids were individually weighed every two weeks throughout the trial. Daily live weight gain (ADG, g/d) values were estimated using regression of weight over time. ADGs were also estimated on the basis of the amount of energy ingested or nitrogen retained by the kids as described Salah *et al*.^23^. Feed intake (offered - refused) was recorded for each kid from Monday to Friday throughout the experiment. The daily dry matter intake of each ingredient and the total intake of DM, OM, NDF, ADF, and CP were determined on a daily and metabolic weight basis. The feed efficiency (FE) was estimated for each kid by dividing DLW by its daily diet intake. Total faecal collection and sampling were used for chemical analyses and to determine total tract digestibility. Four measurement periods of total tract digestibility (each one lasting 5 days) were carried out during the experiment: two before and three after the artificial infestation. The total tract digestibility was estimated by subtracting feed excreted/feed intake from feed intake.

Blood was sampled every week, using EDTA-coated tubes (Becton Dickinson, Plymouth, UK) from each kid using the jugular vein puncture method. The number of circulating eosinophils was measured according to the method described by Dawkins *et al*.^24^. Eosinophils were counted using a Malassez cell counter. PCV was estimated using a capillary microhaematocrit (centrifuged for 5 min at 12,000 rpm). The same day, faecal samples were collected to determine the FECs using the McMaster method as described in Ceriac *et al*.^22^.

Four weeks post-infection, five kids from each infected group were slaughtered to recover and count worms^25^ as described in Ceriac *et al*.^22^. The liver, kidney, and abomasum wall were observed to identify necrotic damage and gastritis, which are indicators of the eventual toxicity of diets provided. The prolificacy of female worms was estimated by calculating the ratio between the daily excretion of eggs (averaged over the 5 days prior to slaughter) and the number of worms counted in the abomasum.

### Laboratory analyses

The dry matter content of feed, refusals, and faeces was established by drying until a constant weight was obtained using a forced draught oven at 60 °C. Samples of the diets, refusals, and faeces were dried under the same conditions and milled through a 1-mm screen (Reich hammer mill, Haan, Germany) prior to analysis. OM and nitrogen analyses were performed according to Association of Official Analytical Chemists (AOAC)^26^, methods 923.3 and 992.15, respectively. To determine OM, ashing at 550 °C for 6 h was performed and nitrogen analyses were performed using the Dumas method. Analysis of the nitrogen content of fresh urine samples was performed using the same method used was used to determine the nitrogen content of diets. The crude protein levels were calculated as N × 6.25. NDF, ADF and ADL in the diets and faeces were determined using a sequential procedure (AOAC methods 200.04 and 973.18, respectively, for NDF and ADF + ADL). CTs were CT extracted and quantified as described in Laurent^27^.

### Statistical analyses

Data were analysed to compare nutritional characteristics (intake, digestion, nitrogen balance, ADG, feed efficiency) of diets using the mixed SAS procedure^28^. Differences between means were tested using the pdiff option. Significance was declared at probability levels ≤ 5%. Only data from the pre-infestation period and the first week post-infestation were considered in order to eliminate a possible effect of parasitism on the intake and digestion of the diets. The value one-week post-infection was considered since the impact of infestation remained low within that timeframe. The statistical model used was as follows:

1 $${{\rm{Y}}}_{{\rm{ij}}}=\mu +{{\rm{D}}}_{{\rm{i}}}+{{\rm{T}}}_{{\rm{j}}}+{{\rm{A}}}_{{\rm{k}}}+{{\rm{e}}}_{{\rm{ijk}}}$$

where Y_ij_ is the explained variable, µ is the mean, D_i_ is the diet fixed effect (I = 1–6), T_j_ is the time relative to the date of the artificial infestation (j = 1–2), A_k_ is the random effect associated with the animal (k = 1–55) and e_ijk_ is the residual term. For the nitrogen balance (faecal, urinary and retained nitrogen), the amount of nitrogen ingested was introduced as a covariate in the statistical analysis model.

Animal responses (nutritional and heath indicators) to the infestation were analysed using the SAS mixed procedure (SAS, 2008). Nutritional indicators were analysed only before infection. PCVs and FECs were log-transformed (log (Eosinophils + 1), log (FEC + 1)) before analysis; however, only raw means are reported in data tables. The general model was as follows:

2 $${{\rm{Y}}}_{{\rm{ijk}}}=\mu +{{\rm{D}}}_{{\rm{i}}}+{{\rm{T}}}_{{\rm{j}}}+{({\rm{D}}\ast {\rm{T}})}_{{\rm{ij}}}+{{\rm{A}}}_{{\rm{k}}}+{{\rm{e}}}_{{\rm{ijk}}}$$

Where Y_ijk_ is the explained variable, µ is the mean, D_i_ is the treatment fixed effect (i = 1–7), T_j_ is the time relative to the date of the artificial infestation (j = 1–5), A_k_ is the random effect associated with the animal (k = 1–57), and e_ijk_ is the residual term.

Abomasum worm count was analysed using a randomised design using the general linear model procedure from SAS, which considered diet a fixed effect. The differences between means were tested using the pdiff option.

### Ethical approval and consent to participate

The animals were treated in accordance with the guidelines and regulations for animal experimentation of the French Ministry of Agriculture. The study was conducted in an INRA experimental unit in Guadeloupe who has an Accreditation to experiment (n°A971802) and involved staff trained in experimentation and animal welfare. The protocol (APAFIS#5527-2016050608133139v2) has been validated by the Ministry of National Education, Higher Education and Research under the advice of the Animal Care and Use Committee of French West Indies and Guyana (N°069).

## Data Availability

Essential data and materials have been reported in the paper. The entire study database, owned by the National Institute of Agricultural Research (INRA), is available from the corresponding author.

## References

[1] Charlier J (2018). Mind the gaps in research on the control of gastrointestinal nematodes of farmed ruminants and pigs. Transboun. Emerg. Dis..

[2] Mueller-Harvey I (2019). Benefits of condensed tannins in forage legumes Fed to ruminants: importance of structure, concentration, and diet composition. Crop Sci..

[3] Hoste H (2015). Tannin containing legumes as a model for nutraceuticals against digestive parasites in livestock. Vet. Parasitol..

[4] Archimède H (2016). Potential of tannin-rich plants, *Leucaena leucocephala, Glyricidia sepium and Manihot esculenta*, to reduce enteric methane emissions in sheep. J. Anim. Physiol. Anim. Nutr..

[5] Athanasiadou S, Kyriazakis A, Jackson F, Coop RL (2001). Direct anthelmintic effects of condensed tannins towards different gastrointestinal nematodes of sheep: *in vitro* and *in vivo* studies. Vet. Parasitol..

[6] Archimède H, Bastianelli D, Fanchone A, Gourdine JL, Fahrasmane L (2018). Aliments protéiques dans les systèmes mixtes intégrés polyculture-élevage en régions tropicales. INRA Prod. Anin..

[7] Frutos P, Hervás G, Giráldez FJ, Mantecón AR (2004). Tannins and ruminant nutrition. Span J. Agric. Res..

[8] Méndez-Ortiz F, Sandoval-Castro C, Ventura-Cordero J, Sarmiento-Franco LA, Torres-Acosta JFJ (2018). Condensed tannin intake and sheep performance: a meta-analysis on voluntary intake and live weight change. Anim. Feed Sci. Technol..

[9] Patra AK (2008). A meta-analysis on effects of supplementing low-quality roughages with foliages from browses and tree fodders on intake and growth in sheep. Livest. Sci..

[10] Patra AK (2009). Effects of supplementing low-quality roughages with tree foliages on digestibility, nitrogen utilization and rumen characteristics in sheep: a meta-analysis. J. Anim. Physiol. Anim. Nutr..

[11] Niderkorn V, Baumont R (2009). Associative effects between forages on feed intake and digestion in ruminants. Animal..

[12] Hoste H (2016). Interactions between nutrition and infections with Haemonchus contortus and related gastrointestinal nematodes in small ruminants. Advances in Parasitology.

[13] Ceï W, Salah N, Alexandre G, Bambou JC, Archimède H (2018). Impact of energy and protein on the gastro-intestinal parasitism of small ruminants: a meta-analysis. Livest Sci..

[14] Méndez-Ortíz FA (2019). Impact of gastrointestinal parasitism on dry matter intake and live weight gain of lambs: A meta-analysis to estimate the metabolic cost of gastrointestinal nematodes. Vet. Parasitol..

[15] Coop RL, Kyriazakis I (2001). Influence of host nutrition on the development and consequences of nematode parasitism in ruminants. Trends Parasitol..

[16] Kyriazakis I (2010). Is anorexia during infection in animals affected by food composition?. Anim. Feed. Sci. Tech..

[17] Kyriazakis I, Houdijk J (2006). Immunonutrition: nutritional control of parasites. Small Rumin Res..

[18] Houdijk JGM (2012). Differential effects of protein and energy scarcity on resistance to nematode parasites. Small Rumin. Res..

[19] Min BR, Hart SP (2003). Tannins for suppression of internal parasites. J. Anim. Sci..

[20] Marie-Magdeleine C, Macheboeuf D, Philibert L, Arece Garcia J, Udino L (2018). Various condensed tannins from tropical plants as potential multi-purpose nutraceutic in ruminant feed. Advances in Animal Biosciences.

[21] Paolini VF, De La Farge F, Prevot Dorchies P, Hoste H (2005). Effects of the repeated distribution of sainfoin hay on the resistance and the resilience of goats naturally infected with gastrointestinal nematodes. Vet Parasitol.

[22] Cériac S (2019). Effect of the nutritional status of Creole goats on the density-dependent prolificacy of Haemonchus contortus. Vet. Parasitol..

[23] Salah N, Sauvant D, Archimède H (2014). Nutritional requirements of sheep, goats and cattle in warm climates: a meta-analysis. Animal.

[24] Dawkins HJS, Windon RG, Eagleson GK (1996). Eosinophil responses in sheep selected for high and low responsiveness to *Trichostrongylus colubriformis*. Int. J. Parasitol..

[25] Gaba S (2006). Estimation of abomasum strongyle nematode infections in sheep at necropsy: tentative proposals for a simplified technique. Vet. Parasitol..

[26] AOAC. Official methods for analysis. Association of Official Analysis Chemists, Gaithersburg, MD, USA (2006).

[27] Laurent S (1975). Etude comparative de différentes méthodes d’extraction et de dosage des tannins chez quelques ptéridophytes. (In French). Arch. Int. Physiol. Biochim..

[28] SAS Institute Inc. SAS Language Guide for Personal Computers, Edition 9.1. (2004).

